# Material fingerprinting: predicting human perception of material appearance through psychophysical analysis and neural networks

**DOI:** 10.1098/rsos.250513

**Published:** 2025-11-12

**Authors:** Jiri Filip, Filip Dechterenko, Filipp Schmidt, Jiri Lukavsky, Jan Kotera, Veronika Vilimovska, Roland W. Fleming

**Affiliations:** ^1^ Institute of Information Theory and Automation, Czech Academy of Sciences, Prague, Czech Republic; ^2^ Institute of Psychology, Czech Academy of Sciences, Prague, Czech Republic; ^3^ Department of Psychology, Justus Liebig University Giessen, Giessen, Germany

**Keywords:** materials, appearance, perception, visual fingerprint, features

## Abstract

Digital representation of materials is crucial in fields such as virtual reality, industrial design and quality control. However, predicting human perception of materials from image data is challenging due to the complexity of material appearances and the intricacies of human vision. This study introduces a perceptual representation termed the ‘visual fingerprint’, linking image-based measurements of materials to intuitive, human-understandable attributes. We conducted psychophysical studies using standardized video sequences of 347 diverse real-world materials, including fabrics and wood, selected to encompass a broad spectrum of textures, colours and reflective properties. Sixteen key appearance attributes were identified, and over 110 000 human ratings were collected to map perceptual attributes across material categories. By integrating CLIP-derived image features with a multi-layer perceptron model, we developed a predictive framework for material perception. Our results demonstrate that human judgements of appearance and similarity can be accurately predicted using only two images of a material. This work offers a practical and interpretable approach to material representation, enabling intuitive comparisons and retrievals in applications where material appearance is crucial. The proposed material fingerprint and its prediction directly from image data represent a significant step towards simplifying the understanding and interoperability of material properties in diverse digital environments.

## 1. Introduction

The digital representation of materials is pivotal in various applications, including virtual reality, industrial design and quality control. Accurately predicting the perceived attributes of these materials from a human vision perspective remains a significant challenge. This difficulty arises from the diversity and complexity of material appearances and the rich spectrum of human perceptual inferences. Moreover, any representation of visual properties should be intuitive, providing users with a clear definition of material attributes applicable to comparison or retrieval. We refer to such a representation as the ‘visual fingerprint’ of a material.

This paper aims to achieve this representation through a combination of psychophysical studies, image processing and machine learning methods. First, we identified critical appearance attributes of a diverse set of real-world materials, including fabrics, leather, wood, plastic, metal and paper, to characterize the space of appearances. The samples were selected to cover a broad spectrum of textures, colours and reflective properties and were imaged to produce standardized video sequences, providing a comprehensive overview of material appearances encountered in both everyday life and specialized industries.

**Figure 1.**
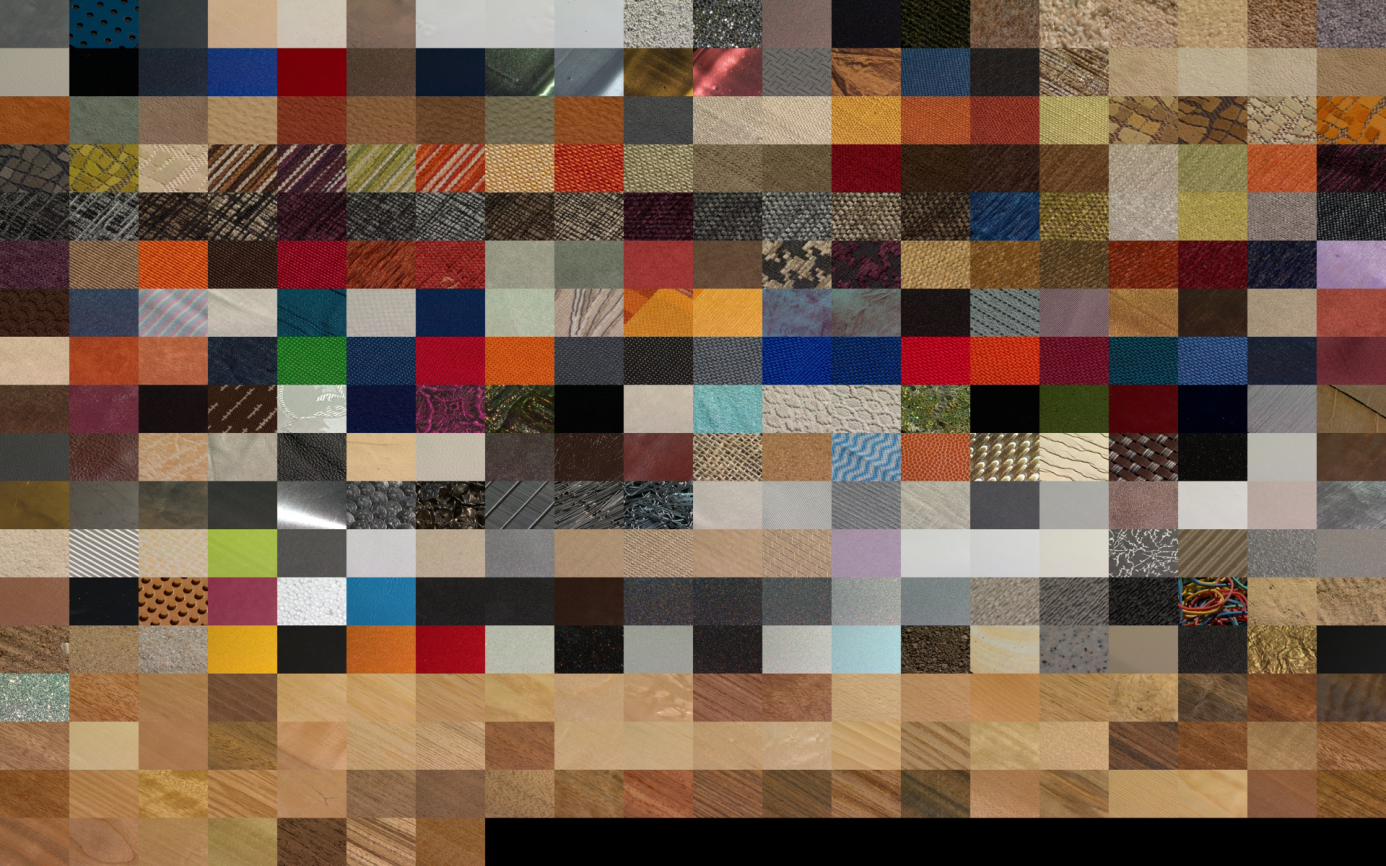
A mosaic of all 347 materials in the study, depicted by a single, non-specular frame (30 out of 60) from the video sequences.

Instead of using only static images, we opted for captured videos showing the genuine material appearance of flat specimens under different viewing conditions [1]. These dynamic material appearance data allowed us to reliably identify 16 key appearance attributes. For these attributes, we collected ratings for 347 materials spanning a wide range of categories (as shown in figure 1) and developed a predictive model of the ratings based on CLIP image features [2] combined with a multi-layer perceptron model trained on the rating data. Our fingerprint provides an interpretive layer that clarifies the relationship between image data and deep learning features for material representation and understanding.

Our findings reveal that combining widely used and easily computed image features with a straightforward machine learning model can predict human judgements of appearance and similarity (visual fingerprints) for a diverse range of materials. This opens new possibilities for perceptually based intuitive and interoperable representations of materials for applications where material appearance is important. Notably, our results demonstrate that visual fingerprints can be effectively deduced from just two images of the material, which may have implications for the efficiency and practicality of material analysis in digital applications.

The primary contributions of our paper are:

— Creation of an extensive public dataset of 347 dynamic material samples, significantly enriching available resources for material studies.
— Identification of 16 crucial perceptual attributes of these materials, providing a foundational understanding of material perception across diverse appearances.
— Collection of over 110 000 ratings from human observers for the 16 attributes across all material samples, offering a rich basis for further analysis.
— Development of a reliable method for predicting rating values using a multi-layer perceptron model by learning a nonlinear mapping from CLIP features, demonstrating the efficacy of machine learning in predicting human perception.

## 2. Related work

Our work relates to human visual perception of material appearance as influenced by illumination and viewing conditions. Specifically, it involves the identification of visual appearance attributes, their approximation through computational features and the variations of these attributes across different material categories.

### 2.1. Perception of texture attributes

Since the beginning of image texture research, scientists have tried to establish a connection between the perceptual texture space and computational statistics. Tamura *et al.* [3] suggested six basic computational texture properties and evaluated their performance in a perceptual experiment on 56 grayscale textures from Brodatz’s catalogue [4]. Rao & Lohse [5] used hierarchical cluster analysis, non-parametric multidimensional scaling (MDS), classification and further analyses to group the same textures and identify a perceptual texture space. They concluded that this space can be represented by three dimensions describing repetitiveness, contrast/directionality and coarseness/complexity. These findings were confirmed by Mojsilovic *et al.* [6] in an experiment with human observers, where they obtained a pattern vocabulary governed by grammar rules, which extended the scope to colour textures.

Later, researchers aimed to link perceptual texture spaces to spaces of computational texture features. Malik & Perona [7] used a vocabulary-based method and a model of human preattentive texture perception, based on low-level human vision, to predict the perception of different textures. Vanrell *et al*. [8] suggested a texton-based four-dimensional texture space with perceptual texton attributes along each dimension. They performed dimensionality reduction of the texton representations to create a low-dimensional behavioural texture space where distances between points represent texture distances. Long & Leow [9] presented an approach to reducing Gabor features, represented by a convolutional neural network, to a four-dimensional texture space.

Researchers have also studied the human perception of specific texture attributes. Padilla *et al.* [10] developed a model of perceived roughness in synthetic surfaces. Pont *et al.* [11] found that observers use texture as a cue for relief depth and that surface roughness can be exploited to increase the realism of standard 2D texture mapping. Ho *et al.* [12] found that roughness perception is correlated with texture contrast.

### 2.2. Perception of textureless material reflectance

Many studies represent material appearance locally or globally by means of the bidirectional reflectance distribution function (BRDF) [13] and its parametric models [14]. Again, scientists have tried to map the model’s parameters to the human perception of materials. Pellacini *et al.* [15] linked parameters of the Ward BRDF model to the perceived gloss of homogeneous surfaces by creating a two-dimensional perceptually uniform space. Similarly, Westlund & Meyer [16] extended the Phong, Ward and Cook-Torrance BRDF models to a parameterization that allows a more perceptually uniform manipulation of the models’ parameters. Matusik *et al.* [17] captured and psychophysically evaluated large sets of BRDF samples, forming the MERL BRDF database, and showed that there are consistent transitions of the perceived properties between different BRDFs. They analysed whether the samples possess any of the 16 predefined perceptual attributes and used the participants’ characterizations to build a model in both linear and nonlinear embedding spaces. This manifold is then used for editing/mixing between the measured BRDFs. Wills *et al.* [18] analysed 55 BRDFs from the MERL BRDF database in an extensive experiment with 75 participants assessing glossiness and used MDS to construct a low-dimensional embedding allowing perceptual parameterization of an analytical BRDF model. Te Pas & Pont [19] showed that changes in surface BRDF and illumination are often confounded, but adding complex illumination or 3D texture improves visual matching. Ramanarayanan *et al.* [20], in a series of psychophysical experiments, presented the concept of visual equivalence by characterizing conditions under which warping and blurring of the illumination maps and warping of the object’s surface yield synthetic images that are visually equivalent to the reference solutions.

Later, Serrano *et al.* [21] psychophysically analysed isotropic BRDFs from the MERL database [17] to identify smooth and intuitive material appearance transitions between different visual attributes. Sawayama *et al.* [22] created a dataset of synthetic images with variable illumination and geometries and conducted perceptual experiments discriminating materials on one of six dimensions (gloss contrast, gloss distinctness of image, translucent vs. opaque, metal vs. plastic, metal vs. glass and glossy vs. painted). They concluded that material discrimination depended on the illuminations and geometries and that the ability to discriminate the spatial consistency of specular highlights in glossiness perception showed larger individual differences than in other tasks. They also demonstrated that the parameters of higher-order colour texture statistics can partially explain task performance.

Lagunas *et al.* [23] gathered observers’ similarity judgements of synthetic images depicting objects with varying materials, shapes and illuminations and trained a deep learning model to measure the similarity in appearance between different materials, which correlates with human similarity judgements and outperforms existing metrics. In follow-up work, Serrano *et al.* [24] collected a large-scale dataset of perceptual ratings of predefined appearance attributes (glossiness, contrast of reflections, sharpness of reflections, metallicness, lightness) for combinations of material, shape and illumination, to analyse the effects of illumination and geometry on material perception. The collected dataset was used to train a deep learning architecture predicting perceptual attributes that correlate with human judgements. Finally, Subias & Lagunas [25] proposed a single-image appearance editing framework based on generative models that allows intuitive modification of the material appearance of an object by increasing or decreasing high-level perceptual attributes describing such appearance (e.g. plastic, rubber, metallic, glossy, bright, rough, and the strength and sharpness of reflections).

### 2.3. Perception of digital representations of textured materials

Extending perceptual analysis to general textured materials introduces a variety of non-local effects such as inter-reflections, masking, shadowing and subsurface scattering. These effects add visual realism but make experimental work substantially more complex. Jarabo *et al.* [26] conducted perceptual experiments to investigate the visual equivalence [20] of rendered images for different levels of bidirectional texture function (BTF) [27] filtering and found that blur in the spatial domain is less tolerable than in its angular counterpart. They analysed whether BTF datasets possess properties such as *high-contrast*, *granular* and *hard*, among others. Deschaintre *et al.* [28] introduced a novel dataset that links free-text descriptions to various fabric materials. The dataset comprises 15 000 natural language descriptions associated with 3000 corresponding images of fabric materials, obtained from observers in the form of free-text descriptions of fabric appearance. By analysing the data, the authors identified a compact lexicon, a set of attributes and key structures that emerge from the descriptions explaining how people describe fabrics. Such annotated image data were used to train a large language model to create a meaningful latent space for fabric appearance, appropriate for material retrieval or automatic image annotation. Finally, Filip *et al.* [1] analysed the perceptual dimensions of 30 wood materials in the form of video stimuli by means of a combination of similarity and rating studies and compared them to basic image statistics. In follow-up work [29], Filip & Vilímovská performed a rating study and linked the rating data with computational statistics, demonstrating the extent to which computational statistics can be used to characterize visual properties on an additional test dataset.

### 2.4. Visual perception across material categories

Recent studies have also analysed material appearance perception as a function of material type. Fleming *et al.* [30] presented an extensive analysis of human perception of materials. In their first experiment, participants judged nine perceptual qualities, while in their second experiment, observers assigned 42 adjectives describing material qualities to six classes of materials. The authors found that the distributions of material classes in the visual and semantic domains are similar and concluded that perceptual qualities are systematically related to material class membership. In a follow-up study, Tanaka & Horiuchi [31] analysed human ratings of the same perceptual qualities as a function of visual information degradation. The authors concluded that general perceptual quality decreased with image-based reproduction, perceptual qualities of images decreased when using their grayscale variant, and perceptual qualities of *hardness* and *coldness* increased when image resolution was reduced. As an alternative to assigning intuitive attributes to materials, some researchers used material image features encoding material-specific characteristics. Schwartz & Nishino [32] introduced the concept of visual material traits, encoding the appearance of characteristic material properties by means of convolutional features of trained patches, to identify local material properties in material recognition tasks. In follow-up work, researchers discovered a space of locally recognizable material attributes from perceptual material distances by training classifiers to reproduce this space from image patches [33,34]. Later, Schwartz & Nishino [35] avoided a fixed set of attributes by proposing a method for deriving material attribute annotations based on probing human visual perception of materials by asking simple yes/no questions comparing pairs of small image patches. This method can be integrated into the end-to-end learning of a material classification CNN to simultaneously recognize materials and discover their visual attributes.

Various aspects of human perception of material appearance have been extensively studied in the past. What sets our study apart from previous work is that (1) our prediction of interpretable appearance attributes is derived from user studies rather than from non-interpretable visual features and (2) we use videos of a diverse range of real-world materials, showing a continuous loop of rotating samples to provide a much richer visual impression than commonly used static or synthetic stimuli. In this work, we build on our previous research on attribute identification [36] and extend it to the automatic prediction of these attributes from image data.

## 3. Method overview

**Figure 2.**
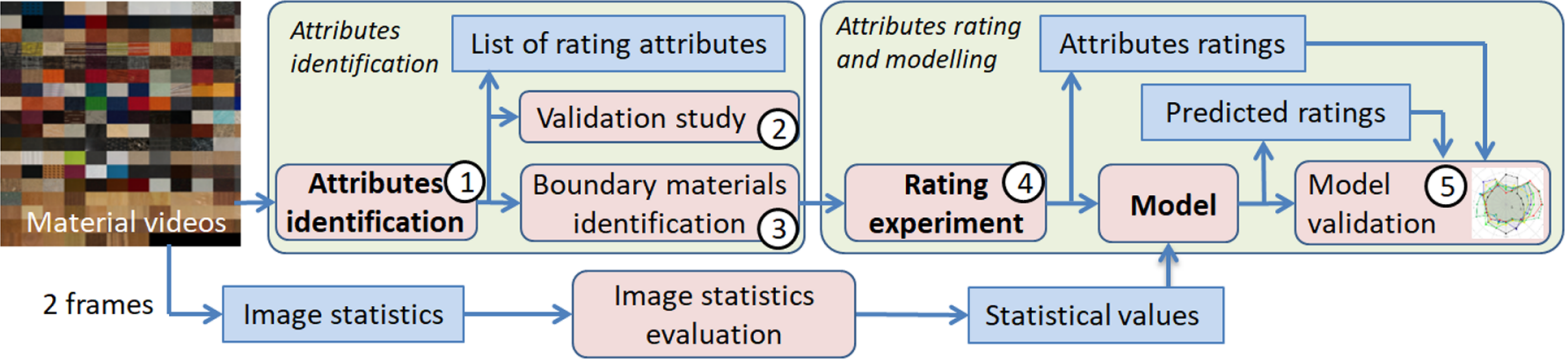
Material characterization by five psychophysical studies to obtain a *visual fingerprint* for 347 material videos and prediction of those attributes with an image-computable machine learning model. Studies and modelling are marked red, resulting data are marked blue.

The entire process of material characterization using a limited set of intuitive features is depicted in figure 2. First, we captured material videos. Then, we identified a set of intuitive perceptual attributes (Studies 1 and 2) and their anchor scales (Study 3). In the following, we obtained attribute ratings for all materials (Study 4), yielding a ‘visual fingerprint’ for each video. To generalize beyond our stimuli, we predicted individual fingerprints with an image-computable model and evaluated its performance in a validation study (Study 5) and in a real-world task using a new set of material photographs.

## 4. Material data capturing

### 4.1. Materials selection

We collected 347 physical material samples, with a focus on capturing a broad variety of visual appearances but also the most common material categories. Although the potential pool of textured materials is vast, we prioritized those for which additional appearance measurements are available, such as benchmark materials from the UTIA BRDF database [37] and the [38] collection [38]. For many material categories with spatially homogeneous appearances, such as metal, plastic and paper, individual materials can be relatively accurately represented using parametric reflectance models [14]. Here, we focused on more visually complex materials exhibiting non-local physical effects like shadowing, masking or subsurface scattering, so that the majority of materials in our selection were woods and fabrics that vary strongly in appearance due to different fibre types [1] and thread weaving patterns [37]. Also, for the remaining categories, we focused mainly on material samples with non-homogeneous structures. Our final selection consisted of 347 physical samples from the material categories of fabric (157), wood (67), coating (30), paper (23), plastic (17), metal (14), leather (11) and others (28) (see figure 1).

### 4.2. Dynamic stimuli capturing

To take into account the role of real-world illumination and the interactions between lighting and object geometry in the estimation of material properties [39,40], we created dynamic stimuli. Specifically, for each material sample, we produced a video sequence showcasing the material’s non-specular and specular characteristics by a slow rotation. These sequences featured close-up views of approximately 42 × 42 mm areas of the samples, captured using the UTIA goniometer [41]. In line with industry standards [42], we maintained a constant polar angle of 45 degrees for both the camera and the light source, varying only the azimuthal angle of the camera to facilitate more rapid measurements. Each sequence commenced with the light and camera azimuthal angles differing by 90 degrees, followed by a 90 degree camera movement, resulting in a final difference of 180 degrees between the azimuthal angles probing specular reflection of the material. Comprising 60 image frames of resolution 632 × 412, each 4 second sequence was played in reverse order after completion, creating an 8 second continuous loop that effectively illustrates the dynamic behaviour of the ‘rotating’ material sample (cf. electronic supplementary material, video S1).

## 5. Selection of main perceptual attributes

### 5.1. Study 1: attributes identification

First, we identify the key visual attributes for describing the appearances of material videos in our dataset (also see figure 2).

**Figure 3.**
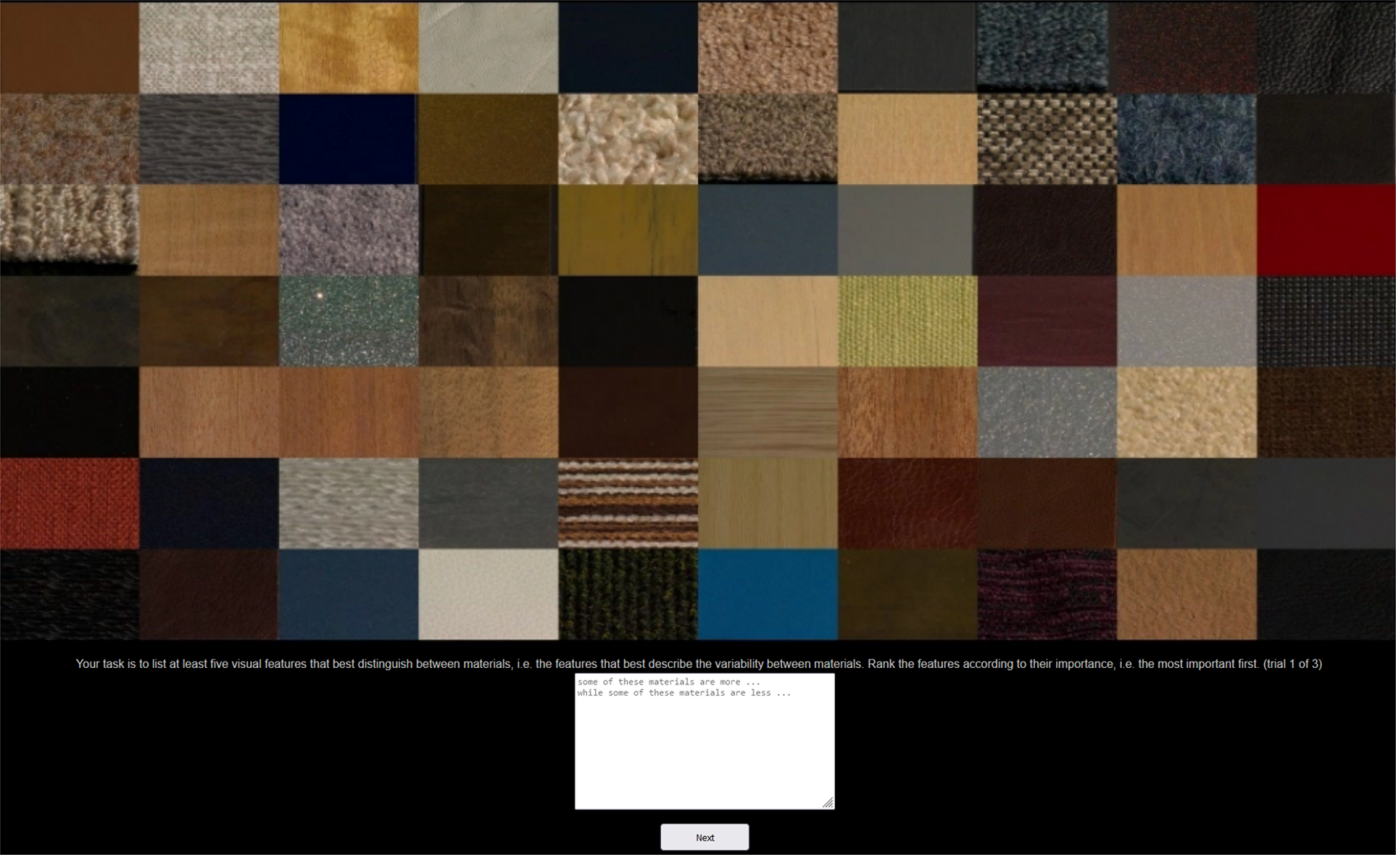
An example of one video frame from a trial of Study 1.

For the online free naming study, we created three arrangements of 70 material videos each, randomly selected from our full dataset; 32 participants were asked in three trials to type at least five most visually distinguishing features that they thought distinguished best between the materials in each arrangement and rank them in the order of their importance. Each participant responded to all three arrangements in different trials (see figure 3). We collected a total of 451 text responses from 32 participants; the mean study duration was 2.8 minutes (SD = 1.8). For all psychophysical studies reported in this paper, including the present one, participants were recruited via the online platform Prolific. We restricted the participant pool to native English speakers with normal or corrected-to-normal vision and no known colour vision deficiencies; however, no formal tests for colour vision or visual acuity were administered. Participants received compensation of £8 /hour for their participation.

**Figure 4.**
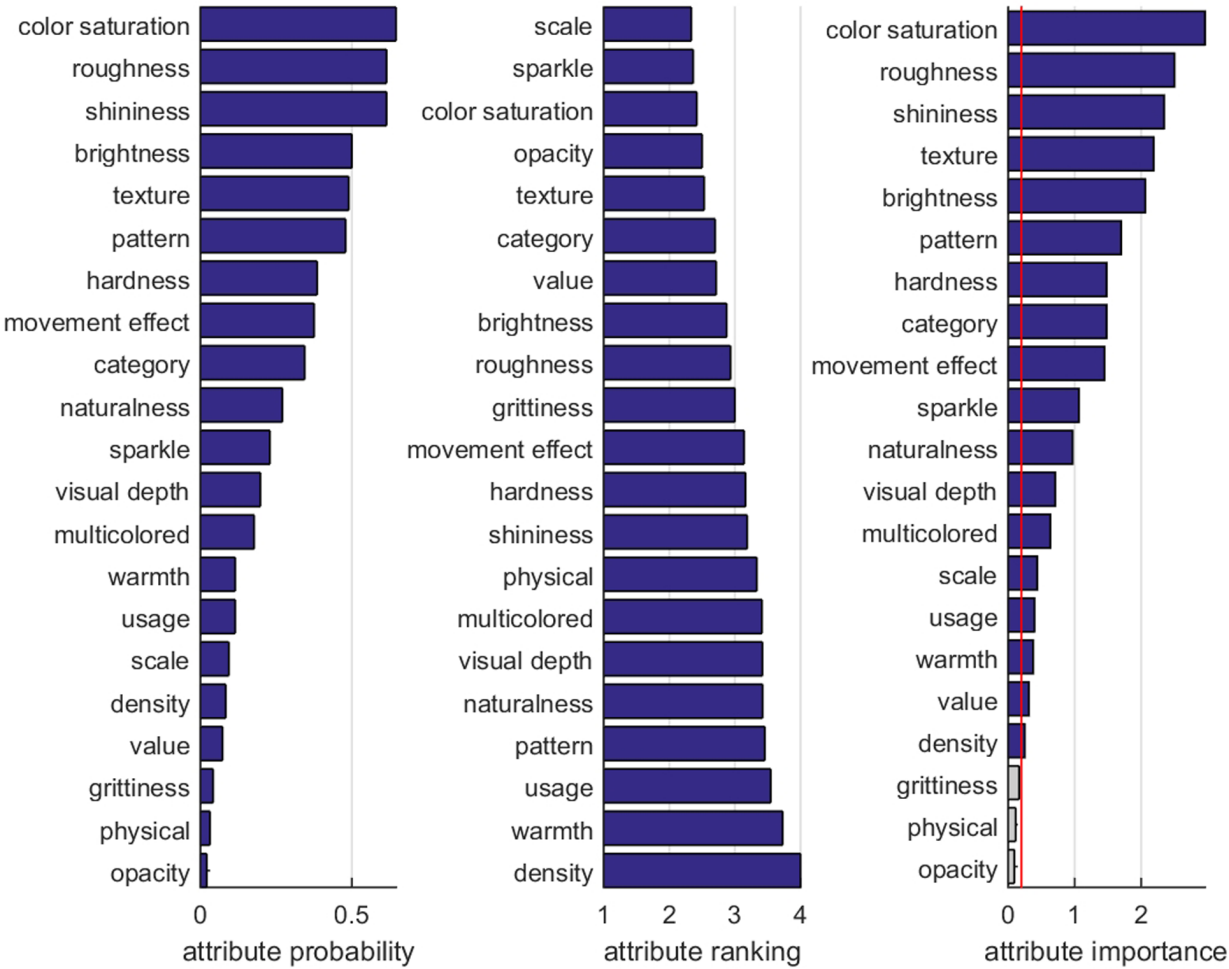
Attribute statistics obtained from the free naming experiment: attribute probability (left), ranking (centre) and the combined importance of each attribute (right).

By manually grouping synonyms and equivalent terms into clusters and removing all responses occurring less than twice (eliminating 0.44% of total responses), we obtained a condensed set of 21 visual material attributes (raw responses for each attribute are provided in the electronic supplementary material). Figure 4 shows the probability $a_{p}$ of each attribute (left; calculated across participants and trials), as well as the average rank $a_{o}$ (centre), and the combined attribute importance (right), calculated by $a_{p}\cdot \left(\max(a_{o})-a_{o}\right)$. As a result, the participants described the visual appearances of our material videos by using typical optical attributes such as colour variability, saturation, roughness, brightness, shininess, texture and pattern, but also tactile or cognitive attributes like warmth, hardness, naturalness and attractiveness. As two of the six most frequent terms, *texture* and *pattern* are vague without further elaboration, we replaced them with the more specific attributes *pattern complexity*, *striped pattern* and *chequered pattern*. The clusters *gritty*, *physical* and *opacity* were removed as they were rarely mentioned (less than five responses). We also removed clusters *usage*, *category* describing utilization or class of the material, and *density* was merged with *hardness*. In total, our clustering included 98.5% of all raw naming responses. The resulting final set of 16 perceptual attributes is shown in table 1, together with the boundary (anchor) terms and the instructive questions that were provided to the participants in our following rating study.

**Table 1.** A list of 16 perceptual attributes evaluated in the rating study and their description.

ID	attribute	boundary values	instructive question
1	colour vibrancy	dull, vibrant	how richly coloured is the material, ranging from monochromatic or neutral-coloured materials to vibrantly coloured materials?
2	surface roughness	smooth, rough	how rough is the material, ranging from fine or smooth to coarse or grainy?
3	pattern complexity	plain, complex	how complex are the patterns on the material, ranging from simple to intricate?
4	striped pattern	‹no, pronounced› stripes	to what extent does the material exhibit stripy patterns?
5	chequered pattern	‹no, pronounced› checks	to what extent does the material exhibit chequered patterns?
6	brightness	dark, bright	how bright is the material, ranging from dim or subdued to bright or luminous?
7	shininess	matt, mirror	how shiny is the material, ranging from dull or non-reflective to highly reflective?
8	sparkle	none, sparkling	to what extent does the material exhibit sparkling and glittery effects?
9	hardness	soft, hard	how hard is the material, ranging from soft or plush to firm or rigid?
10	movement effect	none, extreme	to what extent does the appearance change due to camera movement?
11	pattern scale	fine, large	how large are the pattern elements, ranging from fine-grained or uniform to large or blotchy patterns?
12	naturalness	manmade, natural	how natural is the material, ranging from man-made to natural origin?
13	thickness	flat, thick	how deep is the material structure, ranging from flat or thin to thick?
14	multicoloured	single, many	how multicoloured is the material, ranging from a single or uniform colour to colourful or many colours?
15	value	cheap, luxurious	how valuable is the material, ranging from low-cost or cheap to extravagant or luxurious?
16	warmth	cold, warm	how warm is the material to the touch, ranging from cool or cold to pleasant or warm?

### 5.2. Study 2: attributes validation

As the clustering of attributes might have been subject to experimenter bias, we performed a second study where we asked six participants to cluster all 451 valid text responses into the obtained 16 attributes (see table 1). Overall, the inter-rater agreement was notably high (Fleiss’s kappa score of 0.786), and for 198 out of 451 responses (43.9%), all six raters reached a unanimous decision. For 254 (56.3%) of responses, at least three raters agreed, and for 396 (87.8%) responses, at least two raters did. As a consequence, the naming responses of 16 out of 32 participants in the free naming study fit completely into the derived rating attributes, while the divergence for the remaining 16 participants varied in a range between 5.6% to 26.7%.

## 6. Attributes rating

### 6.1. Study 3: boundary material identifications

**Figure 5.**
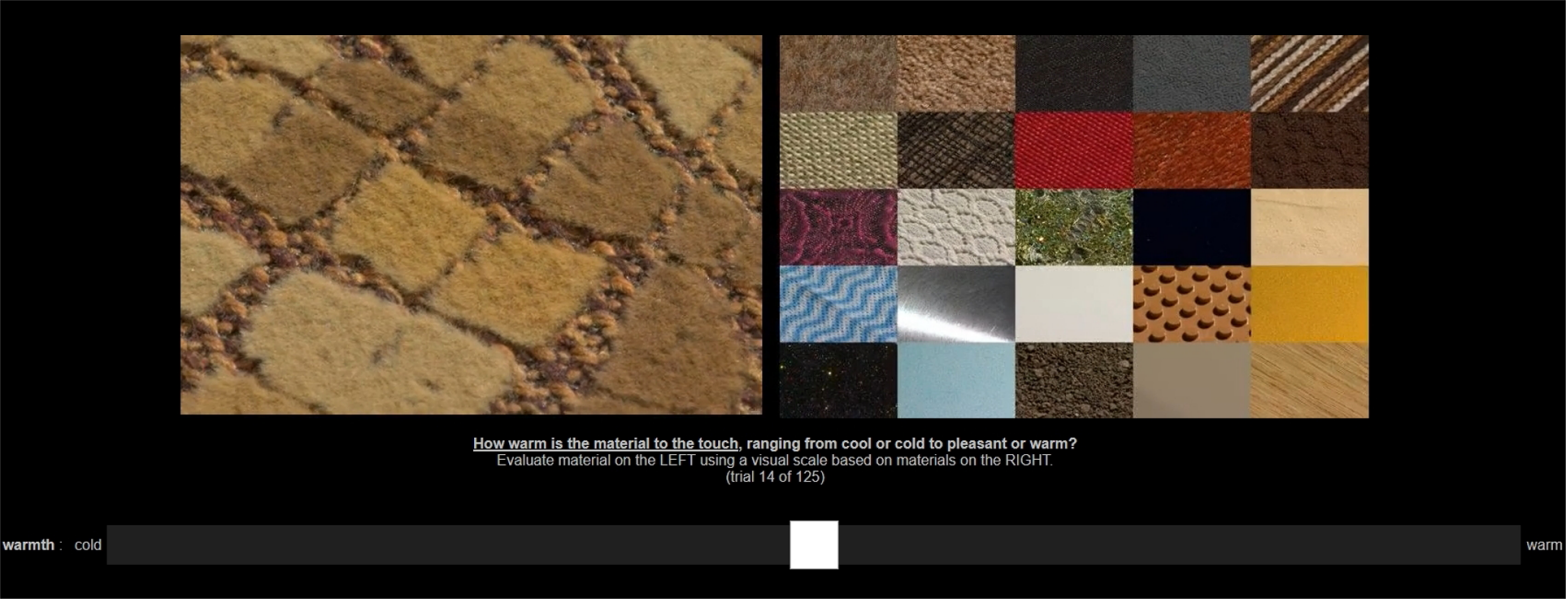
An example trial of the rating experiment with the stimulus on the left and the anchor materials on the right (both were videos, depicted here by a single frame).

To create a representative visual anchor for the rating study, we asked nine online participants to pick from the three arrangements of 70 material videos those materials with the lowest and the highest value of a specified visual attribute (e.g. which of the materials displays the highest level of brightness?). Participants completed 96 responses each (3 arrangements × 16 attributes × 2 boundary anchors). On average, 3.6 (out of 9) participants identified the same video as expressing the lowest value of an attribute and 2.8 participants for the highest value. After removing multiple occurrences (resulting from a video being picked for more than one attribute), we obtained 25 materials that were used as anchors in the following rating study (figure 5).

### 6.2. Study 4: rating experiment

In each trial of the online rating experiment, we showed a material video on the left, together with the arrangement of 25 anchor boundary material videos on the right. For each perceptual attribute, we showed all 347 material videos in random order, and participants responded to the respective question (right column in table 1) with a slider (figure 5). The anchor materials were identical for all tested videos and attributes.

**Figure 6.**
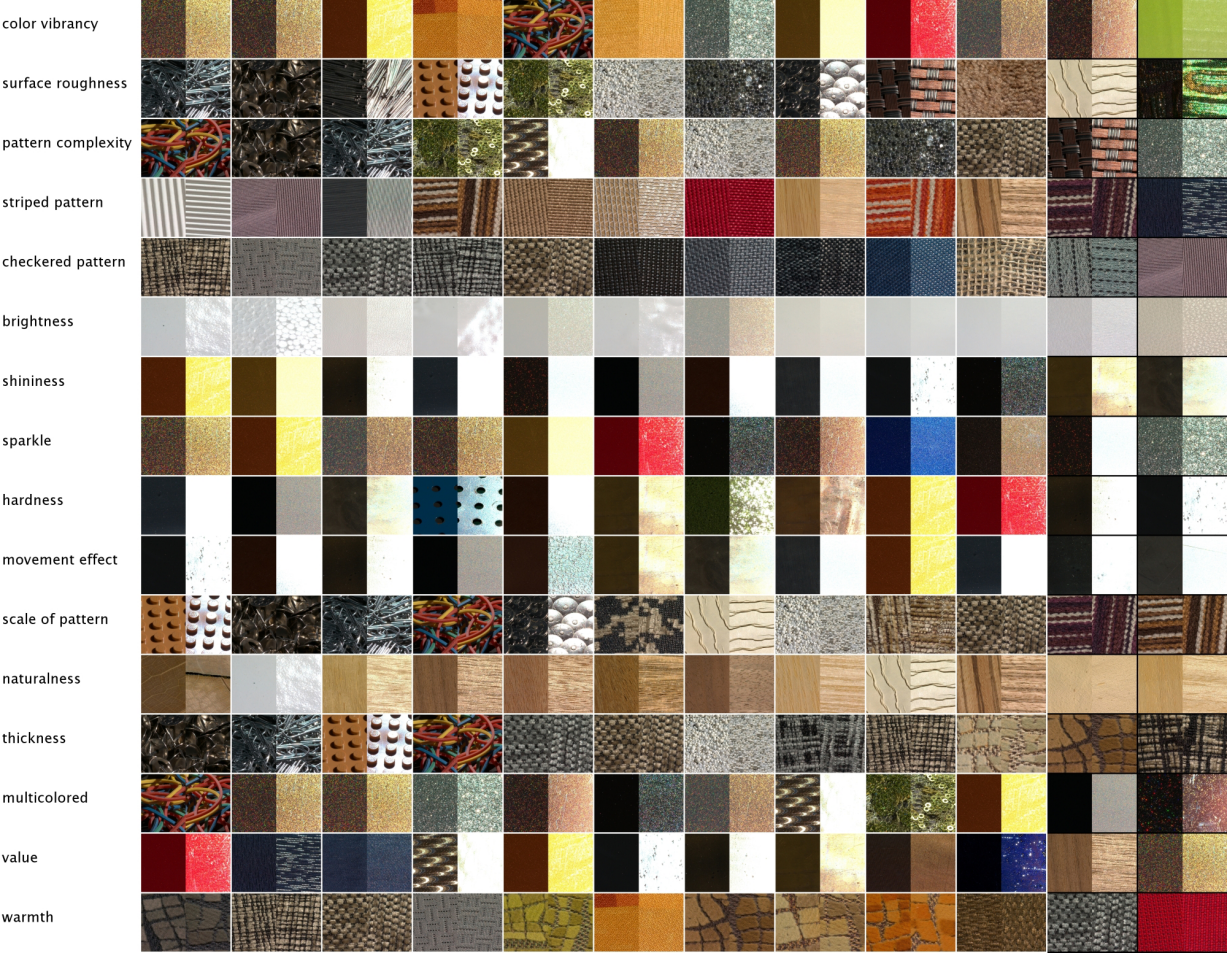
Rank ordering of materials based on their rating values for individual attributes, with the highest value on the left and decreasing values to the right (only showing the 12 highest-rated videos, each depicted by one half of a specular and non-specular frame).

We collected a total of 111 040 ratings (20–24 participants per attribute). After normalizing the data at participant level by Z-scoring, we computed mean rating scores for each material video across all participants. Then, we excluded participants’ ratings from the analysis when the correlation between their material ratings for an attribute with the mean material ratings across all participants for that attribute was negative (removing on average 1.5 participants per attribute). The remaining data were averaged across participants to obtain mean ratings for all 16 attributes and 347 materials. Figure 6 shows the resulting rank ordering of materials for individual attributes.

### 6.3. Material similarity from attribute ratings

We tested a number of different metrics to evaluate the similarity between two materials in terms of their attribute ratings, e.g. L1, L2, correlation, f-divergences and their combinations. Since metrics based on a single computational feature tended to favour either absolute differences (e.g. L1, L2) or relative similarity (e.g. correlation, divergences), we opted to evaluate combined metrics. The authors’ closest visual matches for all pairs across different materials were obtained for a weighted combination of Pearson correlation (R) and L1-norm, where the first term accounts for relative similarity between individual attributes, while the second term compares difference between amplitude of two sets of attributes:


(6.1) $$\begin{array}{lcr} & d(V_{1},V_{2})=\alpha R(V_{1},V_{2})+(1-\alpha )\left[1-\frac{1}{2n}\sum_{i}|V_{1}(i)-V_{2}(i)|\right]. & \end{array}$$

where n is length of attribute vector, i.e. 16, α allows the user to prefer either proportional similarity of attribute values (α=1) or favour minimal absolute distance (α=0) between vectors of ratings. In our experiments, we used α=0.5.

### 6.4. Rating results

**Figure 7.**
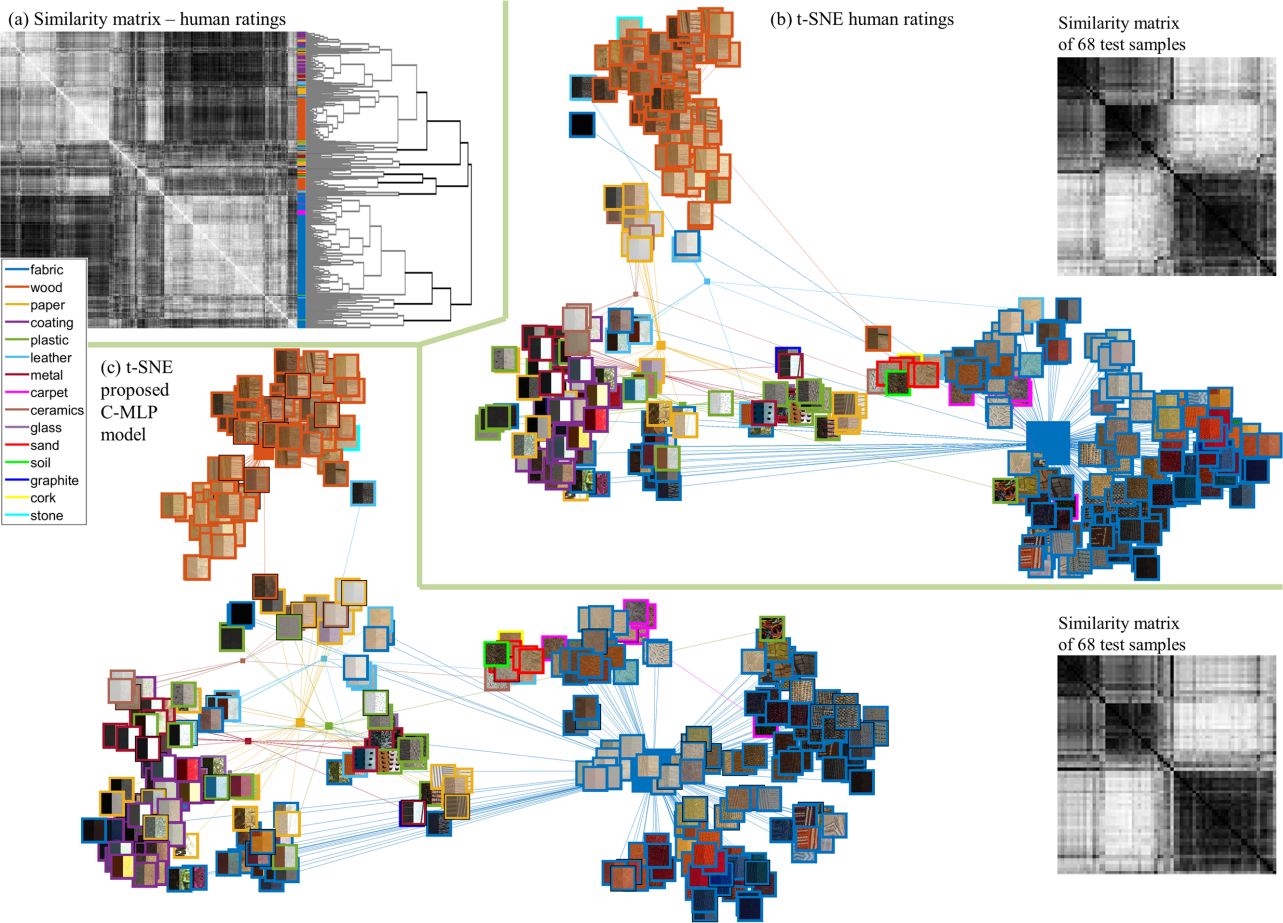
(a) Similarity matrix between all 347 materials with corresponding dendrogram. (b) Similarity of 347 material samples based on human attribute ratings visualized as two-dimensional embedding using t-SNE. (c) Visualization of the proposed C-MLP model (SM) using t-SNE. Ground truth material categories are color-coded; in the t-SNE plots, category means are plotted as coloured boxes with the size depending on the number of samples. (b) and (c) include the similarity matrices for the 68 test samples obtained from the ratings and the proposed C-MPL model.

We visualize the similarity between material samples using t-distributed stochastic neighbour embedding (t-SNE) [43] (figure 7b). For the classes of *wood*, *fabric*, *carpet* and *coating*, we find coherent clustering according to the ground truth category, while we see considerable overlap for other classes. This follows from a high variability in appearance of samples within those classes (and therefore a high variability in attribute ratings), for example, metal sheets vs. pins from the same material (also see figure 7a).

## 7. Visual material fingerprint

**Figure 8.**
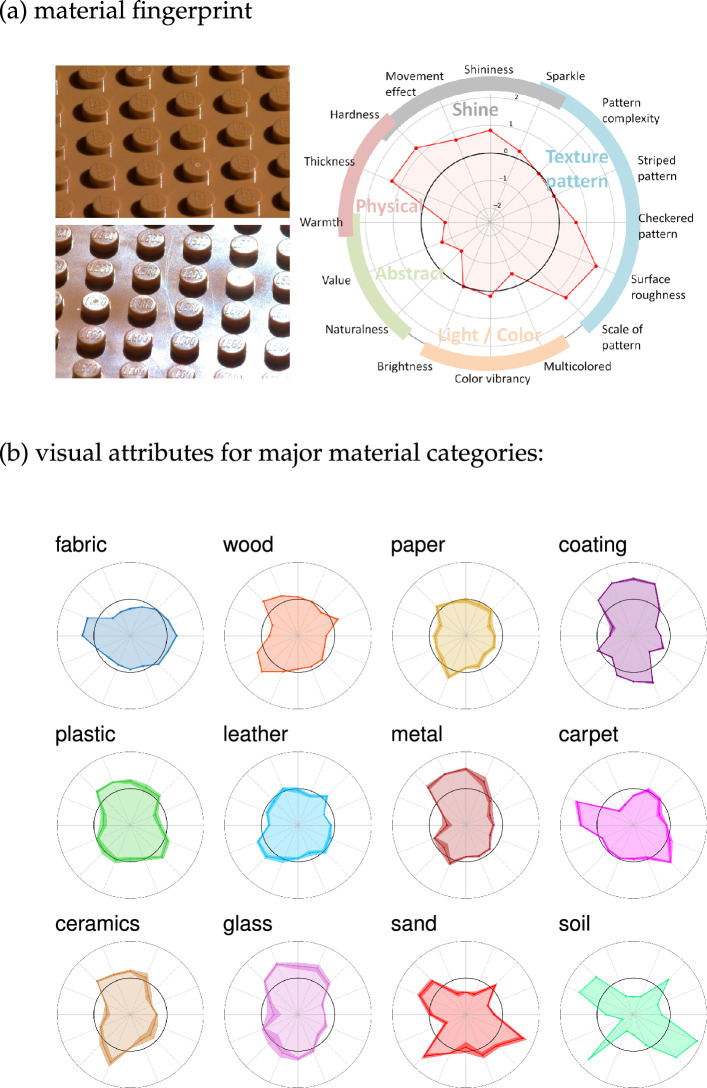
(a) An example of a plastic material (for non-specular and specular conditions) and its visual fingerprint obtained from the rating study, (b) Average fingerprints for 12 major material categories, with the thickness of the plot’s contour indicating the standard error demonstrating variability in the attribute ratings.

The material attributes determine a unique visual signature of a material’s appearance that can be visualized in a polar plot (figure 8a). The azimuthal ordering of attributes is based on their relationships, forming five clusters loosely related to *gloss*, *texture and pattern*, *light and colour*, and both *physical* and *abstract* properties. High values are closer to the plot’s boundary, low values are closer to its centre. Figure 8b displays the median attribute rating values across samples in 12 major material categories from our dataset. This highlights the similarities and differences between the fingerprints of different categories: for example, fabrics and carpets are both characterized as thick and warm, whereas coatings and metals are distinctively not, but rather as hard and shiny.

## 8. Human ratings prediction

In this section, we investigate the possibility of predicting the ‘visual fingerprint’ computationally. Specifically, we propose a method to predict human ratings of visual attributes directly from photographs of material samples. This approach consists of two stages, for which we explored several possibilities. First, we compute a set of intermediate image features, referred to as the ‘image-computable model’, and then we train a classifier to predict human ratings from these features.

### 8.1. Image-computable representation of material images

We tested several sets of image features computed from the material images. Our experiments revealed that a single image from the video sequences did not provide sufficient information about the material’s visual properties, particularly for shiny materials or those exhibiting high dependence on illumination or viewing angle. Therefore, for feature computation, we use two frames from the material videos, corresponding to different observation angles: one for non-specular and one for near-specular behaviour. However, the ideal specular angle, where light is reflected directly into the camera, is not suitable for shiny materials, as the corresponding image frame is often saturated. To address this, we offset the azimuthal angle from the ideal specular angle by approximately 6°.

*Deep learning features*. The main proposed model is motivated by the work of Kaniuth *et al.* [44], who presented an image-computable model of perceived similarity and demonstrated that the deep features of pretrained vision models perform similarly to humans in assessing visual similarity. Inspired by their results, we used the CLIP model (Contrastive Language-Image Pre-training, [2]) as a feature extractor. Specifically, we used the ViT-B/32 backbone with a 224 px input image size and a 512-dimensional output feature vector. Each of the two video frames from a material was first resized to 250 DPI, corresponding to 256 px on the training set, and then centr-cropped to 224 px before being processed by CLIP. The output feature vectors were concatenated, resulting in a 1024-dimensional image-computable model vector. In the results, this model is denoted by the prefix C.

*Statistical features*. In addition to deep features, we also experimented with two different sets of hand-crafted features based on image statistics.

The first set used texture synthesis image statistics introduced by [45], which are prefixed by T in the results. Since these features enable realistic image synthesis, it is conceivable that they are also suitable descriptors of the visual properties of the material. We used three pyramid levels, three orientations and a spatial neighbourhood of 7 × 7 pixels for both video frames and colour channels separately. After removing features with zero values, we ended up with 447 features per channel, resulting in a 1341-dimensional feature vector.

Additionally, we extended the compact set of features that we have successfully used in previous work to find mappings between material images and their human ratings [29], prefixed by S in the results. These 14 features (28 for both frames) consist of up to third-order image statistics, energy of frequency bands, measures of directionality, number of dominant colours and pattern type. More details are provided in the electronic supplementary material, section S10.

### 8.2. Mapping from computational image features to human ratings

In the second stage, we need to establish the mapping between the intermediate image-computable model and the 16 attributes of human ratings (i.e., the visual fingerprint) of the material. For this, we explored two inference methods, as described below.

*K-nearest neighbour model (2NN)*. For each query material sample, we identified k materials in the training dataset that have the closest (in the $L^{2}$ sense) image-model features and predict the perceptual ratings by linear interpolation of the ratings of these samples. We obtained the best performance for k=2, and we denote this method as 2NN.

*Multi-layer perceptron model (MLP)*. We trained a small, fully connected neural network to learn the mapping between the intermediate feature representation and the human ratings. We split the dataset of 347 materials into 80% training and 20% test samples, resulting in a 279/68 split. The test samples were selected randomly for each material category to ensure that all material categories were present in the test set. We adjusted the model size for each of the three image-computable models to achieve the best performance. Specifically, the layer dimensions used were 1341-256-64-32-16 for the texture synthesis features (T), 28-16-16-16 for the statistical features (S) and 1024-512-512-16 for the CLIP features (C). We used ReLU activations in all layers except the output layer. During training, we applied standard augmentations such as random crop, rotation (except for the statistical features, which are inherently rotation-invariant), small scaling (up to 5%) and small perturbations of the azimuthal angle (up to 2.5${,}^{\circ }$). We also tested how the selection of test samples affects the training process by different test sample selection strategies (samples the most distant from the category mean, the closest to the mean and random selection). Across all strategies, we observe that the training loss remains similar throughout the entire MLP training process, indicating that the choice of test samples has minimal impact on training error (see electronic supplementary material, section S12). Test loss is notably higher for atypical samples, but it does not vary significantly between typical and randomly selected test samples.

### 8.3. Results

We tested different variants of image features (T, S, C) and inference methods (2NN, MLP), but only the best-performing are reported in the paper. All computations were done in Python and PyTorch, and implementation details including fingerprint models and inference codes are provided on the project website (https://osf.io/tkzh4).

*Correlation analyses*. We compared the predictive performance of four models (C-2NN, T-MLP, S-MLP and C-MLP) on 16 perceptual features across 347 materials. The second column of table 2 compares the explained proportion of variance in the similarity matrices computed from model predictions and human ratings; the third column shows the proportion of variance in the human ratings that is explained by the model predictions $R_{D}^{2}$; while the fourth column shows the mean average errors. A Friedman test revealed a significant effect of model type ($p=5.96\times 10^{-10}$), with mean $R_{D}^{2}$ values and 95% confidence intervals indicating a clear performance hierarchy: C-2NN (0.437), T-MLP (0.680), S-MLP (0.757) and C-MLP (0.939). Wilcoxon signed-rank tests confirmed that each model significantly outperformed less expressive ones (all p<0.001), with C-MLP clearly outperforming all others.

**Table 2.** Models comparison based on explained variance and error. Reported are the proportions of explained variance from the similarity matrix ($R_{S\;M}^{2}$) and from raw rating data (averaged across attributes) ($R_{D}^{2}$), along with mean average error. Bold figures indicate the best values across the tested methods.

method	_ $R_{S\;M}^{2}$ _	$R_{D}^{2}$	MAE
(a) all data (347 samples)			
T-MLP: texture synthesis	0.753	0.680	0.285
S-MLP: image statistics	0.835	0.757	0.251
C-2NN: CLIP model	0.712	0.437	0.381
C-MLP: CLIP model (proposed)	**0.943**	**0.939**	**0.117**

(b) test samples only (68 samples)			
T-MLP: texture synthesis	0.687	0.394	0.378
S-MLP: image statistics	0.755	0.610	0.305
C-2NN: CLIP model	0.823	0.454	0.375
C-MLP: CLIP model (proposed)	**0.893**	**0.800**	**0.220**

The results remained consistent when we analysed only performance on 68 test materials not used for the models’ training: C-MLP again outperformed all other models (mean $R_{D}^{2}=0.800$), while C-2NN remained the weakest (0.454). Notably, T-MLP showed substantially lower generalization performance (0.394) compared to the full dataset, suggesting potential overfitting or limited expressivity. The Friedman test remained significant ($p=2.23\times 10^{-9}$), and Wilcoxon comparisons confirmed that C-MLP significantly outperformed all alternatives (all p<0.001). These results reinforce the superior generalization capability of the C-MLP model even on a reduced, seen sample. Further details, including confidence intervals, are provided in electronic supplementary material, section S11.

**Figure 9.**
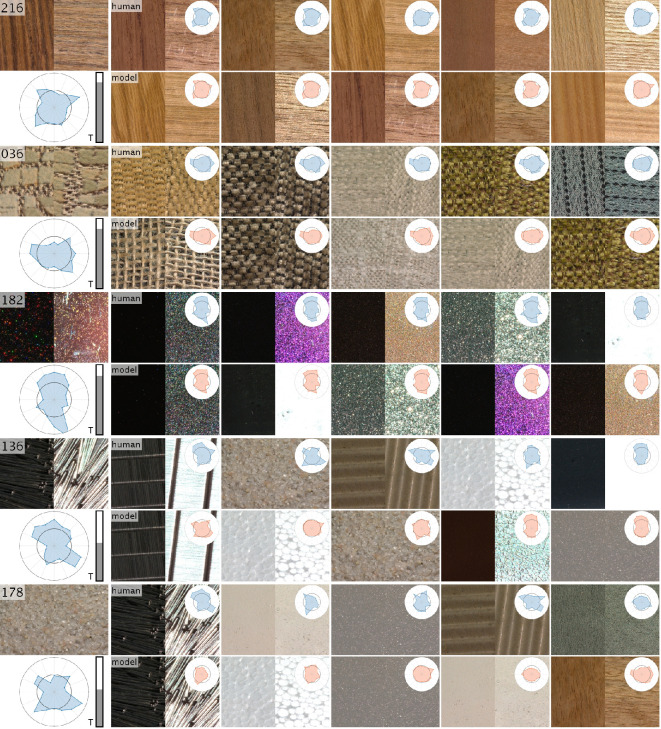
Example test material samples (left) with their visual fingerprints (below), followed by the five most similar samples according to human ratings (blue fingerprints) and model predictions (red fingerprints), identified by the strongest correlation coefficients between attributes. We show results for material samples of different typicality, indicated by the grey bar plot. All samples are depicted by one-half of a specular and non-specular frame.

**Figure 10.**
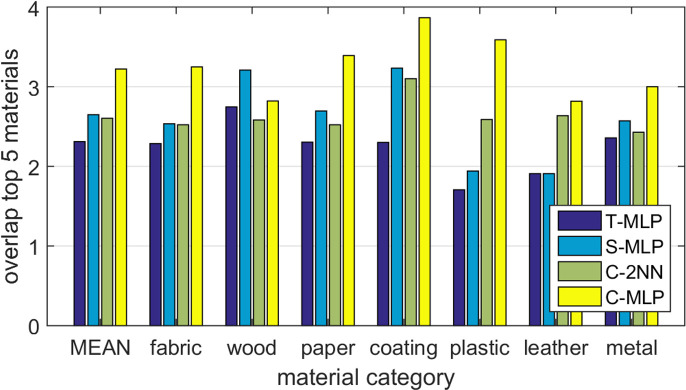
(a) Overlap of the top five material images across attributes for model predictions versus human ratings in the test set. (b) Percentage of observers that for a given material sample preferred the closest four material samples were selected based on either the human ratings, the proposed C-MLP model, or a random selection.

*Retrieval overlap*. To further compare all tested models, we also evaluated the overlap between the top five material images in the test set for each attribute based on the model predictions versus the human ratings as shown in figure 9. As a result, the C-MLP model again clearly outperformed other models for all material categories but wood (figure 10a), with an average overlap of 3.2 of 5. Based on these results, we consider C-MLP as the best performing model.

*Samples proximity analysis*. When visualizing the C-MLP model in a t-SNE plot (figure 7c), we observed a very similar pattern as in human ratings with woods and fabrics being clearly separated from other category clusters (see electronic supplementary material, section S7 for an MDS visualization). In figure 7b,c, we also plot similarity matrices computed from model predictions and from human ratings for 68 test samples for visual comparison.

**Figure 11.**
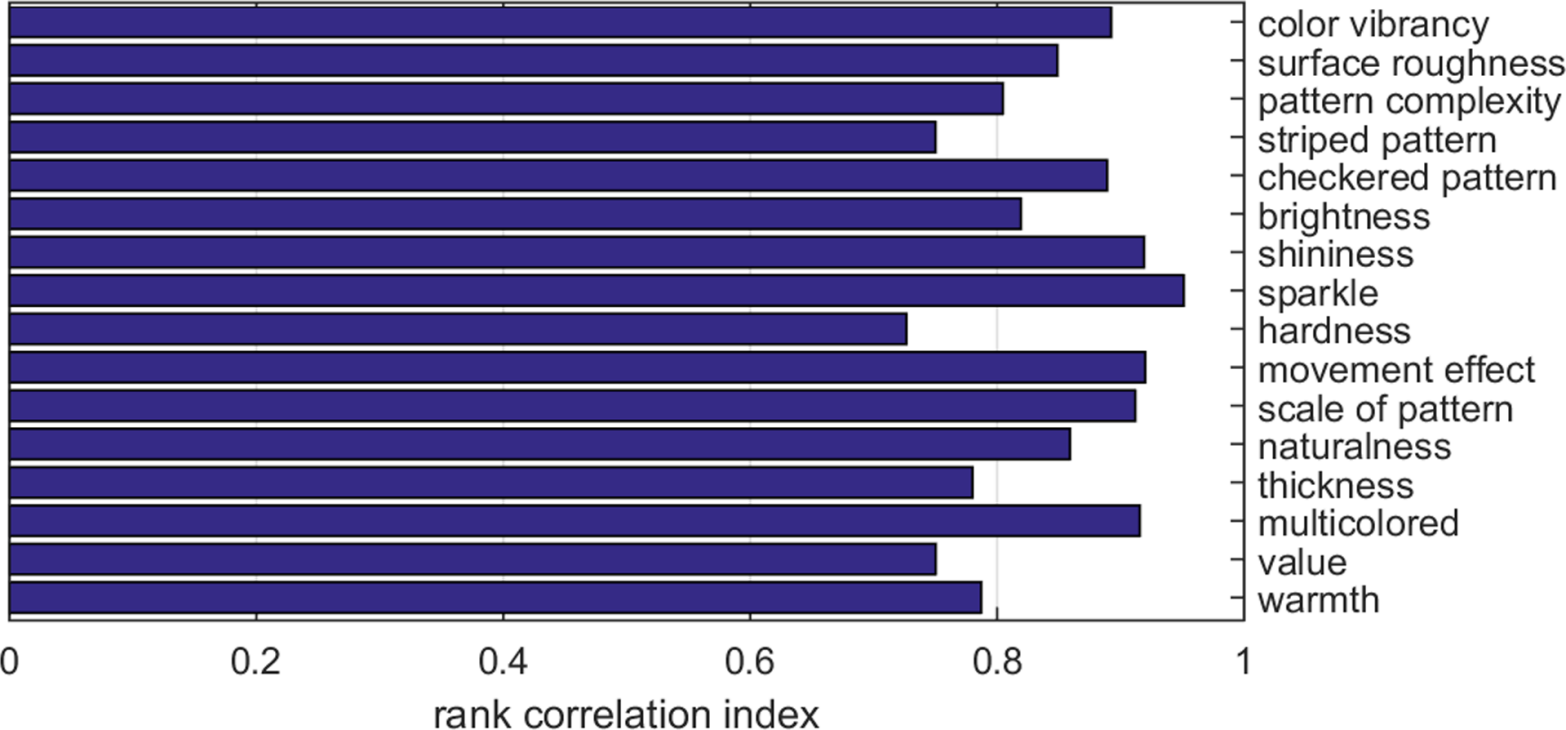
Spearman rank correlation index (RCI) for the order of the first 100 images when ranked by human ratings or model predictions, separately for each attribute.

*Ranking analysis*. For each attribute, we also computed the Spearman rank correlation (RCI) to measure the overlap between the ordering of the first 100 images according to the human ratings versus the model predictions (figure 11). The largest overlap (0.9 < RCI < 1.0) was obtained for ‘optical’ attributes like *shininess*, *sparkle*, *movement effect*, *scale of pattern* and *multicoloured*. In contrast, attributes related to physical or more abstract material characteristics obtained lower scores (0.7 < RCI < 0.8), such as *hardness*, *value*, *thickness*, *warmth* and *striped pattern*.

### 8.4. Study 5: validation experiment

**Figure 12.**
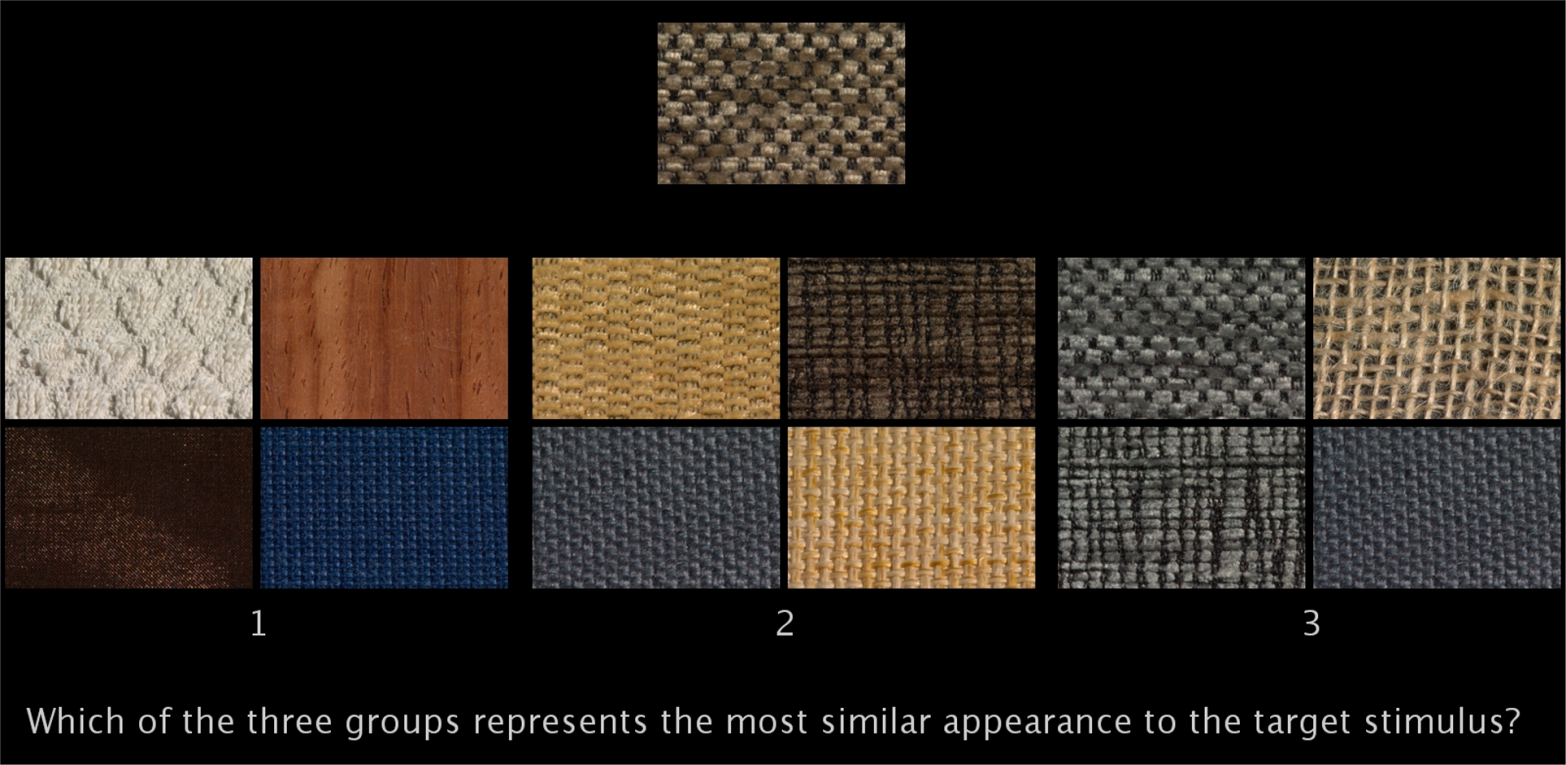
An example of one video frame from a trial of the validation experiment.

To validate our C-MLP model in terms of predicting valid perceptual judgments, we presented each material test sample together with three groups of four samples (figure 12). One group contained the four closest material samples selected based on human ratings, one group those selected based on model predictions and one group four randomly selected samples; 22 online observers evaluated all test samples in 68 trials and were asked: *Which of the three groups represents the most similar appearance to the target stimulus?* The order of groups on the screen was random.

**Figure 13.**
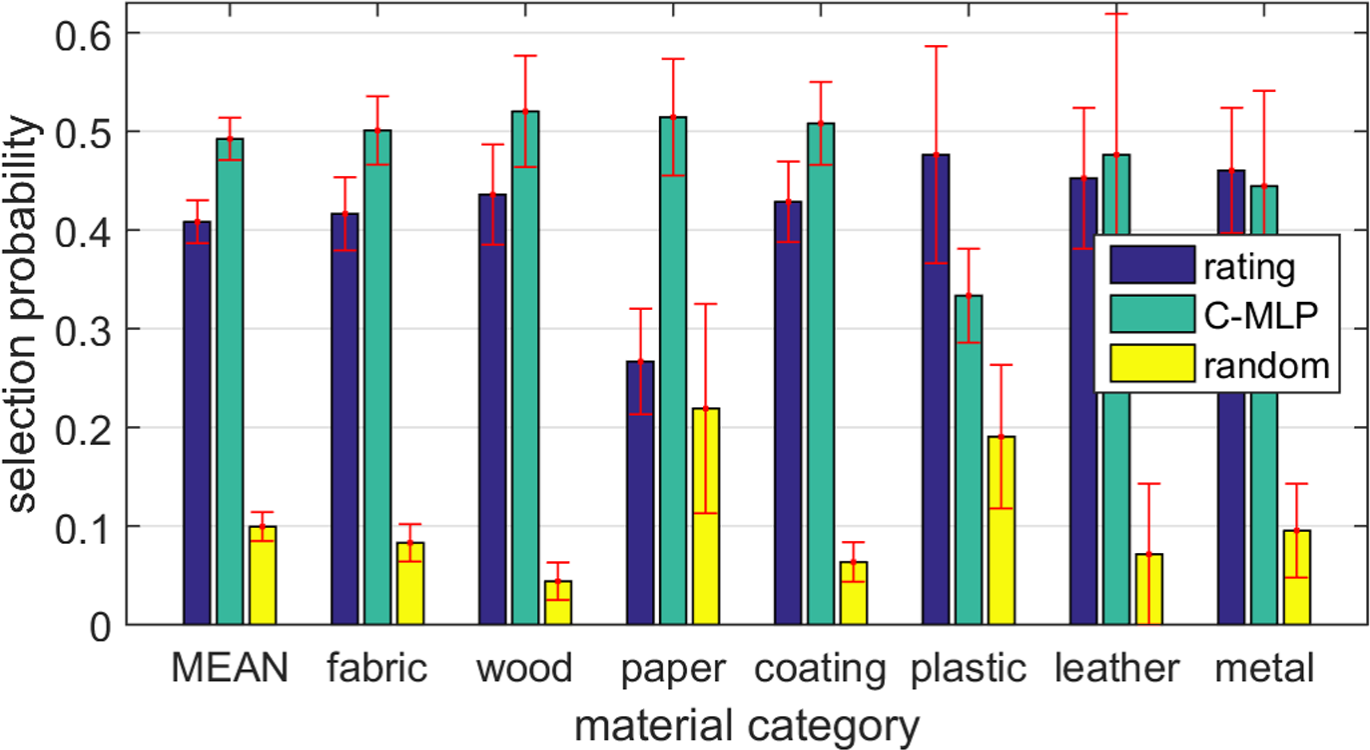
Percentage of observers who, for a given material sample, preferred the closest four material samples for each material category selected based on either the human ratings, the proposed C-MLP model or a random selection. The error bars represent standard errors.

Figure 13 presents averages computed across both participants and materials. For each material, we determined the proportion of participants who selected each of the three options: human ratings, model prediction or random selection and then averaged these proportions over all 68 materials. On average, 41% of observers preferred the group representing the human ratings, while 49% favoured the group based on the winning C-MLP model. A breakdown of the results by individual categories shows that our model is rated more favourably than the baselines in all categories except plastic and metal. In the case of plastic, this may be attributed to the wide variability in its visual appearance, which limits the number of similar materials available for comparison (see electronic supplementary material, section S2). This is further supported by the increased preference for randomly selected materials in this category.

### 8.5. Material retrieval

In the following, we can also use the distances between visual fingerprints (6.1) to identify materials with similar appearance. In figure 9, we plot for exemplary test material samples (left) the five most similar samples according to both human ratings (blue fingerprints) and model predictions (red fingerprints). The results suggest that the retrieval is highly effective when samples of similar appearance are available in the dataset, for example, for wood (216), fabric (036) or coating (182). However, even for more sparse categories, the retrieval identifies plausible samples, for example, for pins (136) or sand (178). We can quantify the similarity to other samples or ‘typicality’ of each sample by averaging across the distances to the 10% most similar samples (figure 9, grey bar plots). The retrieval results for all materials are provided in a separate document in the electronic supplementary material.

### 8.6. Material retrieval in the wild using smartphone shots

**Figure 14.**
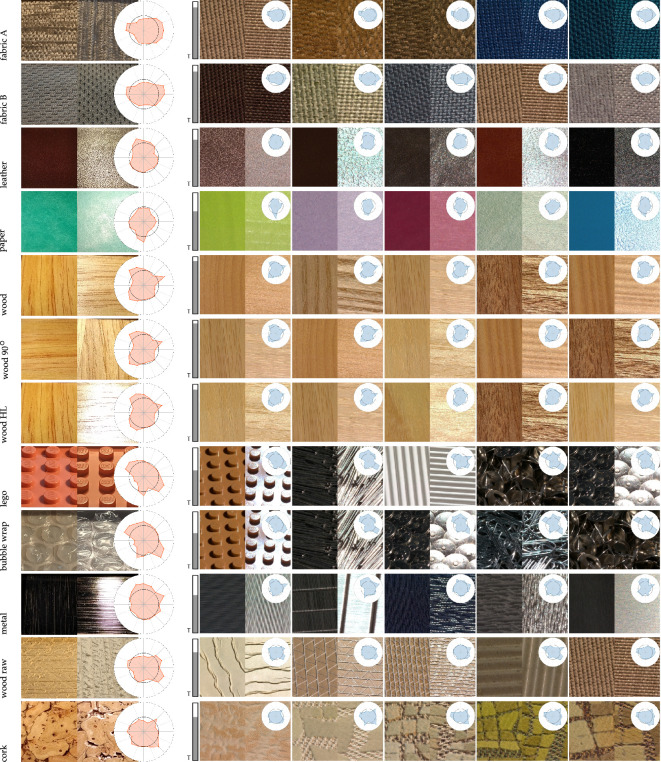
Material retrieval of the five closest material samples from our dataset for two smartphone close-up shots (non-specular, near-specular) of materials in the wild. A typicality of the material samples is indicated by the grey bar plot.

To test our material retrieval method in the wild, we took smartphone close-up shots of different material surfaces, each under non-specular and near-specular conditions (light opposite the camera or on the left side of the sample). Then we cropped the images to obtain an area of interest 26 × 26 mm, rescaled to 51 × 2512 pixels, and used our C-MLP model to predict the visual fingerprints. We used standard lens settings and a capturing distance of 30 degrees. The captured images are not corrected for perspective, white balance or colour. Figure 14 dataset (grey bars) and the five most similar material samples from the set. We obtained very good matches from our dataset for standard materials like fabric, leather, paper and wood. To measure the effect of orientation, we included rotated shots of the same wood material, resulting in very similar predicted fingerprints and retrieval. We also tested less typical materials, resulting in the retrieval of material samples with similar visual features. More examples are provided in the electronic supplementary material, section S9.

## 9. Discussion

In this paper, we introduce (1) 16 intuitive perceptual material appearance attributes that can be used for effective material identification and retrieval and (2) a computational model for their prediction directly from material image data.

### 9.1. Advantages of visual fingerprinting

Our method allows material retrieval according to perceptual appearance attributes (visual fingerprint) which therefore align with human observers’ intuitions about material similarity. The model is trained on > 100 000 human rating responses and can estimate human appearance judgements of any novel material from image data. As a result of capturing materials for both non-specular and specular behaviour, our experiments also suggest some degree of invariance to orientation, the exact illumination and viewing angles.

Our visual fingerprinting method enables a range of practical applications in material research and industry. It supports the creation of private material libraries, allowing designers and engineers to catalogue in-house samples and maintain consistency across teams. In manufacturing, it helps identify visual differences and similarities between production batches or product lines, supporting quality control and appearance validation. When selecting materials for a new product, users can quickly retrieve samples that visually match a reference, streamlining decision-making. The method also allows users to explore large material collections based on perceptual attributes, which is particularly useful in design workflows where appearance plays a critical role.

### 9.2. Computational and memory demands

We compared the performance of all inference models with respect to their complexity, by computing the Akaike information criterion for the 68 test samples (table 3, first column). The results show [46] the lowest complexity for S-MLP and C-2NN as they rely on very small sets of parameters when compared to the texture synthesis T-MLP and CLIP-based C-MLP models that both have a more complex MLP structure. The compared methods also vary in terms of model loading or initialization overhead, inference speed and memory footprint (table 3). The model based on statistics (S-MLP) requires precomputing some data structures for faster inference, while the CLIP-based models require time-demanding loading of models to memory and have larger memory demands. Our results show that after initialization, the CLIP features allow the highest inference speed.

**Table 3.** A comparison of the tested models in terms of the Akaike information criterion (AIC) and the speed of the visual fingerprint inference from material images (including the one-time model loading) and model memory footprints. All tests were done on an Intel Core i7 with 16 GB RAM.

method	AIC	initialization	inference	model
		overheads (s)	speed (s)	size (MB)
T-MLP	667.6	**0.0**	0.62	1.5
S-MLP	2.4	1.6	0.4	**0.004**
C-2NN	**1.6**	1.7	0.07	337.1
C-MLP	666.9	1.7	**0.07**	340.1

### 9.3. Limitations and future directions

*Number of samples*. Our dataset of 347 materials was carefully selected from a portfolio of real-world materials from different categories and is one of the largest sets used in a psychophysical analysis to date. However, the number of samples per category varies, with the most samples for fabric and wood. This might have biassed the model results, which is also suggested by the clustering in figure 7.

*Observation and illumination geometry*. In contrast to previous studies, we used video stimuli, providing observers with rich information on the dynamic appearance of materials under different viewing angles, including both specular and non-specular conditions. However, this is, of course, still a limited subset of all possible lighting-sample-viewer configurations. For example, we had to limit the camera and light trajectories to produce videos of reasonable length. Therefore, we did not potentially account for all specific retroreflective, goniochromatic or anisotropic behaviours that for some materials might result from further changes in viewing and lighting polar angles.

*Sensitivity to exact geometries*. Capturing exact geometries is not always possible; however, the C-MLP model is quite robust for differences up to 15${,}^{\circ }$ as demonstrated on a comparison of images obtained by smartphone camera and comparisons between video frames and BTF data (see electronic supplementary material, section S9). On the other hand, using the same geometries will produce more consistent results as any angular deviation can lead to slightly different fingerprints and material retrieval results (cf *wood* vs. *wood HL* in figure 14). Our experiments demonstrate consistent performance when the camera view remains fixed while the light source moves, making mobile capture more convenient.

*Fixed sample scale*. Although our experiments demonstrate a certain robustness to material scale variations, our current C-MLP model is limited to a fixed resolution and a fixed level of detail of the material surface (sample area of 26 × 26 mm, resampled to a resolution of 512 × 512 pixels). Also, our analysis is limited to materials with relatively fine and stationary textures and cannot describe visual behaviour beyond our sample size, i.e. textures with too low spatial frequencies or too slow gradient changes across the sample.

*Open questions and future directions*. While we tested statistical and deep learning features that proved to predict the human visual encoding of image data well [44,47], there are of course other features which might potentially perform even better. However, we consider this work as a proof-of-concept study, which can be extended in the future by testing different predictive models. To support this endeavour, we have made all stimuli (video and image data), rating responses, code for statistical analyses and the trained C-MLP model available via a public repository. Finally, we provide an online application that evaluates the visual fingerprints of novel materials on demand.

## 10. Conclusions

Through psychophysical studies involving 347 material samples across various categories, we identified a set of 16 material attributes and obtained over 100 000 observer ratings that define a visual fingerprint for each sample. We tested various image features and inference methods and identified a multi-layer perceptron model using deep learning features from a CLIP model as the best-performing approach to map material image statistics to human ratings. Our findings suggest that this model can predict visual fingerprints of material samples, supporting intuitive comparisons between materials and the retrieval of materials with similar appearances from the dataset. We validated the method by obtaining visual fingerprints for real-world materials from close-up smartphone shots. Additionally, we provide users with an online app MatTag offering an intuitive interface between material images and their interpretable perceptual attributes, facilitating efficient organization of materials in various applications.

## Data Availability

The obtained psychophysical data and source codes can be downloaded from https://osf.io/tkzh4/. A mobile application MatTag implementing our method is available at https://github.com/adamstas/material-fingerprint-app. Supplementary material is available online [48].
